# Comparison of Affymetrix Gene Array with the Exon Array shows potential application for detection of transcript isoform variation

**DOI:** 10.1186/1471-2164-10-519

**Published:** 2009-11-12

**Authors:** Kevin CH Ha, Jasmin Coulombe-Huntington, Jacek Majewski

**Affiliations:** 1 Department of Human Genetics, McGill University, Montreal, QC, Canada; 2 McGill University and Genome Quebec Innovation Centre, Montreal, QC, Canada

## Abstract

**Background:**

The emergence of isoform-sensitive microarrays has helped fuel in-depth studies of the human transcriptome. The Affymetrix GeneChip Human Exon 1.0 ST Array (Exon Array) has been previously shown to be effective in profiling gene expression at the isoform level. More recently, the Affymetrix GeneChip Human Gene 1.0 ST Array (Gene Array) has been released for measuring gene expression and interestingly contains a large subset of probes from the Exon Array. Here, we explore the potential of using Gene Array probes to assess expression variation at the sub-transcript level. Utilizing datasets of the high quality Microarray Quality Control (MAQC) RNA samples previously assayed on the Exon Array and Gene Array, we compare the expression measurements of the two platforms to determine the performance of the Gene Array in detecting isoform variations.

**Results:**

Overall, we show that the Gene Array is comparable to the Exon Array in making gene expression calls. Moreover, to examine expression of different isoforms, we modify the Gene Array probe set definition file to enable summarization of probe intensity values at the exon level and show that the expression profiles between the two platforms are also highly correlated. Next, expression calls of previously known differentially spliced genes were compared and also show concordant results. Splicing index analysis, representing estimates of exon inclusion levels, shows a lower but good correlation between platforms. As the Gene Array contains a significant subset of probes from the Exon Array, we note that, in comparison, the Gene Array overlaps with fewer but still a high proportion of splicing events annotated in the Known Alt Events UCSC track, with abundant coverage of cassette exons. We discuss the ability of the Gene Array to detect alternative splicing and isoform variation and address its limitations.

**Conclusion:**

The Gene Array is an effective expression profiling tool at gene and exon expression level, the latter made possible by probe set annotation modifications. We demonstrate that the Gene Array is capable of detecting alternative splicing and isoform variation. As expected, in comparison to the Exon Array, it is limited by reduced gene content coverage and is not able to detect as wide a range of alternative splicing events. However, for the events that can be monitored by both platforms, we estimate that the selectivity and sensitivity levels are comparable. We hope our findings will shed light on the potential extension of the Gene Array to detect alternative splicing. It should be particularly suitable for researchers primarily interested in gene expression analysis, but who may be willing to look for splicing and isoform differences within their dataset. However, we do not suggest it to be an equivalent substitute to the more comprehensive Exon Array.

## Background

Alternative pre-mRNA splicing is a mechanism that allows the production of multiple transcript isoforms of the same gene. While our understanding of splicing has increased over the years, it remains a challenge to carry out genome-wide profiling of transcript isoforms, many of which may play important biological roles, contribute to human phenotypic diversity, or confer susceptibility to complex genetic diseases and cancer [1-3].

In the past, the detection of alternatively spliced genes using 3' targeted gene expression arrays has been limited [4,5]. A number of attempts have been made to predict tissue specific isoform variants using these arrays [6,7]. With the advancement of new isoform-sensitive microarrays, one popular platform to emerge is the Affymetrix Human Exon 1.0 ST microarray (Exon Array), in which 25-mer oligonucleotide probes target exons (with approximately four probes per exon probe set) across the length of the gene. We have previously shown that the Exon Array is effective in characterizing alternative splicing and isoform variation at a genome-wide scale and demonstrated that genetically controlled isoform variation is widespread in human populations [8,9]. In a later study, we compared the Exon Array's performance to that of other standard 3' arrays using the high quality MicroArray Quality Control (MAQC) RNA [10], derived from human brain tissue and a universal human reference, and observed comparable performances between both types of platforms [11]. More recently, Affymetrix has released another whole-transcript gene expression microarray, the GeneChip Human Gene 1.0 ST Array (Gene Array), where a majority of probes are derived from the Exon Array. A study by Pradervand et al. demonstrated, also using MAQC samples, that this platform performs comparably to other 3' arrays [12]. With an average of approximately one to two probes targeting each individual exon (for a total of 764,885 distinct probes) in over 20,000 well-annotated genes, the Gene Array bears a close resemblance to the Exon Array in their design. However, little is presently known on whether the Gene Array expression data can be used to detect alternative splicing and isoform variation.

In this report, we compared the expression data generated by Pradervand et al. [12] and our previous study [11] to examine the inter-platform reproducibility between the Gene Array and the Exon Array. We also developed an approach to examine the Gene Array at the exon level and explored in detail its potential for detecting alternative splicing and isoform variation. We show that while the Gene Array has reduced coverage and is not as comprehensive as the Exon Array, it will provide users the opportunity to maximize the value of their expression data and additionally look for potential alternative splicing events.

## Results

### Gene expression comparison across platforms

We studied the performance of the Gene Array and the Exon Array by comparing previously published experiments performed on the MAQC RNA samples (see Methods). Four technical replicates of brain and reference tissue groups from each platform, for a total of 16 samples, were used in our analysis. Next, probe hybridization intensity values were summarized using the probe logarithmic intensity error (PLIER) method [13] and their respective fold change differences were compared. Here, we use fold change (FC) defined as FC = Expression_Brain_/Expression_Reference _to quantify expression differences between the two tissue groups. In order to facilitate the comparison between the two platforms, we limited our analysis to a subset of genes that had RefSeq annotation and were targeted by both the Gene Array and Exon Array. It should be noted that although the RNA samples were standardized across all replicates, the Gene Array and Exon Array experiments were processed in different labs, and under slightly different protocols. Thus all comparisons reflect both inter-platform and inter-lab variability.

**Figure 1:**
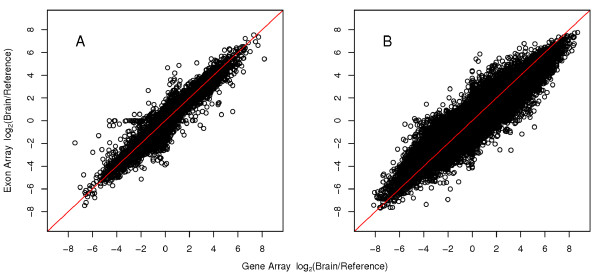
**Comparison of fold changes detected between the Gene Array and Exon Array**. Fold changes (log_2 _transformed) detected by the two platforms are highly correlated at both the (A) gene level (R = 0.94) and (B) exon level (R = 0.91). A background filtering and correction step was applied using an expression cutoff of 30. The red line indicates the x-y diagonal.

We first conducted our comparison at the gene expression level, which the Exon Array has been shown to be able to measure effectively [11,14-16]. Our results demonstrated a highly correlated expression fold change pattern (R = 0.92) on 14,880 genes (data not shown). The majority of discordant genes (i.e. genes in which the Gene Array FC and Exon Array FC are inconsistent) were observed to have very low hybridization levels in both platforms, suggesting that these discordant genes may not be expressed or represent signal below background noise levels. To remove non-expressed genes, we applied filtering criteria and established a background expression cutoff on values summarized by the PLIER algorithm (see Methods). Repeated analyses over a range of threshold values yielded an optimal expression cutoff of 30, resulting in an improved correlation (R = 0.94) on 14,378 genes (Figure 1A). The results of this optimization analysis are illustrated in Additional File 1 - Figure S1A. This analysis also illustrates that a PLIER gene level cutoff of 30 is an optimal background threshold, and that genes with lower expression levels should be treated with caution. Overall, the high concordance of these results shows that the Gene Array and Exon Array perform similarly at the gene expression level. It should be noted that the correlation is quite remarkable, given that the arrays were processed in different labs using different protocols.

### Exon level expression comparison across platforms

The Exon Array is a powerful and comprehensive platform for studying isoform variation because of its ability to summarize probe hybridization intensity values at the exon level [17-19]. In order to enable exon level analysis on the Gene Array, which was primarily designed for gene expression profiling, we modified one of the standard library files employed by Affymetrix expression analysis software such as PowerTools. This file, called the Probe Group File (PGF), defines groupings of probes into probe sets. For the Gene Array, probe sets are defined to approximately correspond to a gene transcript. By contrast, Exon Array probe sets are defined as roughly corresponding to an exon. Modifications to the Gene Array PGF were made such that the probes were grouped to correspond with the Exon Array probe sets. By limiting our analysis to only probes common to both platforms and using only core Exon Array probe sets for high confidence data, we were able to group 409,775 (53.6% of 764,885) probes into 230,074 exon probe sets. The average probe coverage was slightly less than half of the Exon Array (approximately 1.8 probes per exon on Gene Array versus 3.8 probes per exon on Exon Array).

After summarization of probe intensities using the new Gene Array PGF, we compared the exon level expression profiles across the two platforms. As before, probe sets expressed below background levels were removed, first by discarding all exons from genes that were previously removed at the gene level due to lack of detectable expression, followed by repeating the filtering procedure on the remaining exons. Again, a PLIER score of 30 was found to be a suitable threshold score at the exon level (see Additional File 1 - Figure S1B). As expected, the overall correlation of the calculated fold changes for 205,465 exon probe sets (14,236 genes) between both platforms was slightly lower than the gene level correlation (R = 0.91) as shown in Figure 1B. While a small percentage of exons exemplified a two-fold difference in fold changes between platforms (0.9%), the additional variation found in this comparison may be mainly attributed to the greater number of data points. To evaluate the effect of the reduced probe coverage on expression measurements, the 205,465 probe sets on the Gene Array were grouped according to the number of probes targeting each exon probe set (probes per probe set). The number of probes per probe set ranged from one to four probes, where the majority probe sets are targeted by one (n = 93,032) or two (n = 81,135) probes. The correlation of fold changes within each of the four groups showed a subtle improvement in the correlation coefficient with an increasing number of probes (see Additional File 2). Probe sets targeted by one probe and two probes had correlation coefficients of R = 0.90 and 0.92, respectively. In the remaining exons that are targeted by three (n = 20,926) or four probes (n = 10,372), the correlation coefficient only slightly improved (R = 0.92 and 0.94, respectively). The results demonstrate that the Gene Array, despite having fewer probes per probe set, is also capable of profiling exon level expression.

Conversely, we considered the utility of the additional probes on the Exon Array. Here, the Exon Array PGF was modified by removing a randomly selected probe from each probe set with more than one interrogating probe, and then re-summarizing the expression levels. This effectively reduced the average probes per probe set on the Exon Array from 3.8 to 2.8. The step was repeated a second time, bringing the average to 1.9 probes per probe set. At each iteration, the re-calculated fold changes were compared to the full Gene Array fold changes (see Additional File 3). Similar to the results observed with the Gene Array above, the fold changes remained highly correlated, indicating that the additional probes on the Exon Array do not considerably increase sensitivity of expression measurements.

### Comparison of previously detected alternative isoforms

**Figure 2:**
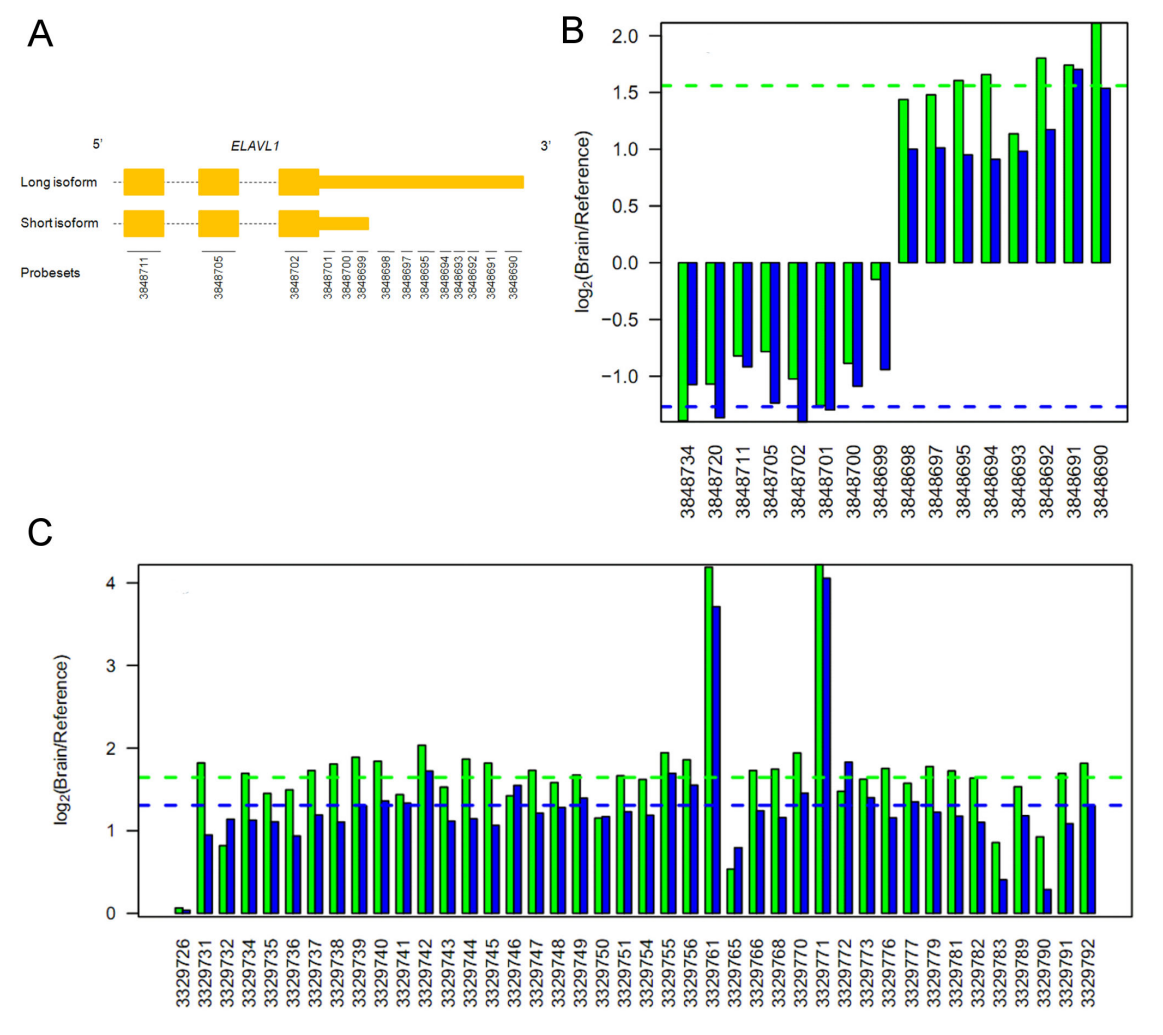
**Comparison of Gene Array and Exon Array expression of isoform variants between brain and reference tissues**. (A) Schematic of the 3' end of the long and short isoform of *ELAVL1*, illustrating an alternative polyadenylation site at the UTR. Exons are indicated in orange, introns as dashed lines, and exon probe sets as solid lines. (B) Expression profile of *ELAVL1 *as measured by both platforms (Gene Array in green, Exon Array in blue), confirming the two isoforms. Exon level log_2 _fold changes are indicated by the vertical bars and summarized by each exon probe set within the gene as indicated on the horizontal axis in 5' to 3' direction. Gene level log_2 _fold changes are indicated by horizontal dashed lines across the gene and coloured accordingly to correspond to each platform. Note the discrepancy in estimating gene expression fold changes for *ELAVL1*. (C) Similarly, an expression profile of *MADD*, illustrating cassette exons at 3329761, 3329771, and 3329783.

We previously characterized genes with tissue specific isoform variants in our Exon Array MAQC data. In particular, the genes *ELAVL1 *and *MADD *were described in detail [11]. To gain insight into whether the Gene Array can make the similar detection calls, we compared our results using these examples. Our previous study showed that *ELAVL1*, located on chromosome 19p13.2, expresses two isoforms due to an alternative polyadenylation site within the 3' UTR region (Figure 2A). The longer isoform was found to be predominantly expressed in the brain while the shorter isoform was expressed in the reference sample. Figure 2B shows a plot comparing the fold changes for *ELAVL1 *at both the exon and gene level. It can be observed that the results at the exon level show a similar fold change pattern in both platforms (R = 0.98) and agree with the previous results. Surprisingly, however, the overall gene level fold changes of *ELAVL1 *do not correspond very well. The overall gene expression was estimated to be higher in the brain by the Gene Array, but higher in the reference group by the Exon Array. This illustrates the difficulty of defining "gene expression levels" in the presence of different isoforms, and the fact that this definition is handled differently by the two platforms.

In the second example we considered *MADD*, located on chromosome 11p11.2, where three alternatively spliced Exon Array probe sets (3329761, 3329771, and 3329783) were previously characterized and supported by RefSeq annotation evidence [11]. Again, at the exon level the Gene Array was capable of demonstrating a similar fold change pattern to the Exon Array (R = 0.91; Figure 2C). Two of the alternatively spliced exons (probe sets 3329761 and 3329771) are expressed at higher levels in the brain, while the third exon (3329783) is more abundant in the reference tissues, as has previously been indicated by the Exon Array data.

### Correspondence of splicing index analyses

The identification of alternative splicing events from Exon Array data has been a major challenge [11], and several approaches have recently been proposed in this area [20-22]. Here, for the sake of simplicity of the comparison across the platforms, we use the most intuitive approaches: probe set level analysis and splicing index (SI) analysis. The probe set level analysis was used to visualize fold changes and to compare the exon expression profiles between the two platforms as shown in Figure 2B and 2C. While effective, this method relies heavily on a manual intervention approach.

**Figure 3:**
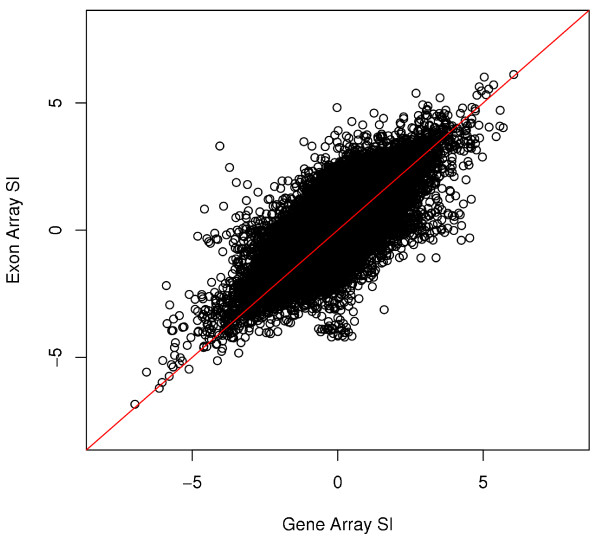
**Comparison of SI values between the Gene Array and Exon Array**. After applying background filtering and correction with a cutoff of 30, the SI values between the two platforms were reasonably correlated (R = 0.61), suggesting that the Gene Array has a potential to detect alternative splicing and isoform variation genome-wide. The red line indicates the x-y diagonal.

To further quantify the correspondence of our results between the two platforms, we calculated the SI value of each exon, here defined as the log_2 _ratio of the normalized index (NI) between the brain and reference, where NI is expressed as exon probe set intensity divided by the gene level intensity [23,24]. The use of SI is also not without its limitations and is discussed in our previous study [11]. In particular, the SI is highly sensitive to noise at the exon and gene level which propagate into the numerator and denominator. For this analysis we retained only genes that were expressed above background levels in both samples since, by definition, only those genes can be considered alternatively spliced across samples. Using the expression value cutoff of 30, we obtained a correlation of R = 0.61 from a total of 163,910 probe sets. Figure 3 shows a correlation plot of SI values between the Gene Array and Exon Array after filtering and background correction. In addition, an intra-platform comparison of SI values is presented in Additional File 4. It can be seen that while the splicing-level analysis is less concordant than gene and exon expression level results, a good correlation was obtained suggesting that the Gene Array has potential capabilities of profiling alternative isoforms.

### Coverage of known alternative splicing events

We next addressed the question of how comprehensive the coverage of each of the microarrays is by determining how many known alternative splicing events, based on EST and mRNA data, are targeted by each platform. Using the Known Alt Events (Alt Events) annotation track from the UCSC Genome Browser (Human March 2006 hg18 assembly) [25], we surveyed the number of documented alternative splicing events that shared overlap based on genomic chromosomal coordinates with Gene Array probes and Exon Array probe sets. In order to obtain a broad overview of each platform's coverage, all Gene Array probes and Exon Array probe sets (core, extended, etc.) were considered. Our analysis showed extensive coverage of the Exon Array of a high proportion of known events across different alternative splicing categories (55,629 out of 74,059 annotated events, 75%; Table 1). The Gene Array, as expected, targets a lower proportion of the annotated alternative events (34,412, 46%). We note that both platforms showed a high coverage of cassette exons (68.0% of total known cassette exons on the Gene Array; 89.1% on the Exon Array). Other categories that were moderately to highly represented include retained introns (40.7% Gene Array; 88.3% Exon Array), bleeding exons (45.9%; 82.5%), alternative 3' splice sites (33.9%; 48.0%), alternative 5' splice sites (39.4%; 57.5%) and alternative promoter usage (39.0%; 76.4%). This comparison provides an estimate of the proportion of all annotated alternative events that could in principle, under best case scenario conditions, be detected by the two platforms.

**Table 1:** Summary of overlap between Alt Events and probes by isoform type

Isoform Type	Total Alt Events	# of Events Overlapped by Gene Array	# of Events Overlapped by Exon Array
Alternative Polyadenylation Site	1,684	41 (2.4%)	229 (13.6%)
Alternative 5' Intron Splice Site	3,634	1,433 (39.4%)	2,088 (57.5%)
Alternative 3' Intron Splice Site	5,332	1,806 (33.9%)	2,558 (48.0%)
Alternative Promoter Site	19,409	7,577 (39.0%)	14,826 (76.4%)
Overlapping Exon	9,876	4,535 (45.9%)	8,149 (82.5%)
Cassette Exon	24,511	16,670 (68.0%)	21,840 (89.1%)
Minor Introns*	182	22 (12.1%)	144 (79.1%)
Retained Intron	5,150	2,095 (40.7%)	4,549 (88.3%)
Strange Intron Ends**	4,281	233 (5.4%)	1,246 (29.1%)

*Total*	*74,059*	*34,412 (46%)*	*55,629 (75%)*

* Intron with AT/AC ends rather than the usual GT/AG

** Intron with ends that are not GT/AG, GC/AG or AT/AC

The total number of Alt Events sharing overlap with Gene Array and Exon Array platforms is summarized in the table above. Results are categorized based on isoform type. Definitions of each isoform category can be found in the Alt Events track description page in the UCSC Genome Browser http://genome.ucsc.edu/cgi-bin/hgTrackUi?db=hg18&g=knownAlt. Note that some Alt Events overlap each other and are considered independently.

### Comparison of efficiency of splicing detection between platforms

**Figure 4:**
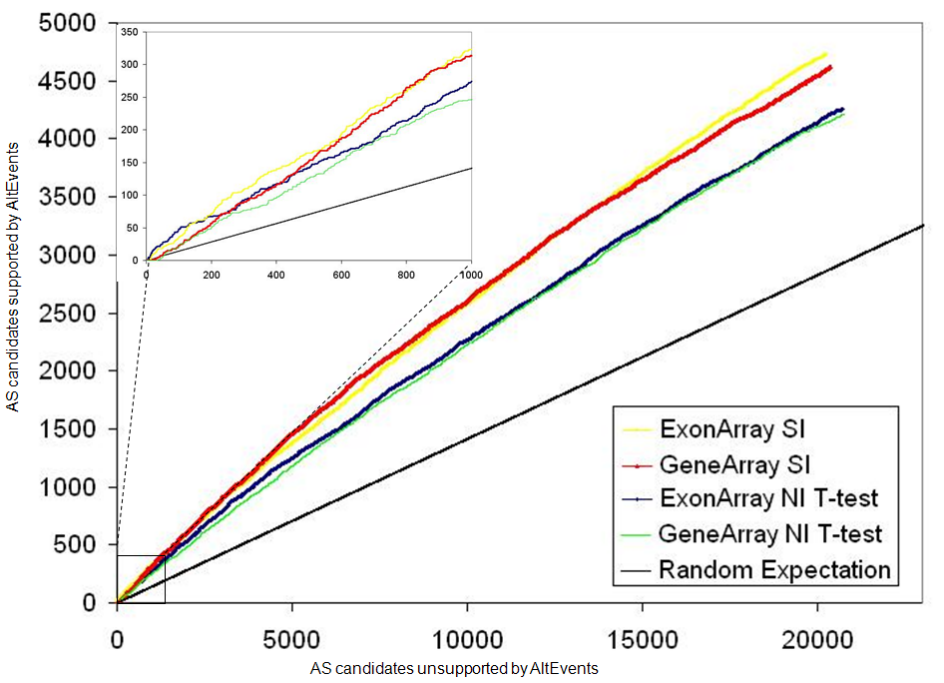
**Agreement with Alt Events for each platform**. This plot illustrates the comparison between the Gene Array and Exon Array in detecting known splicing events (based on the Alt Events UCSC track) as determined using SI and t-statistic on NI. Lines are colour-coded according to the figure key. The subplot provides a zoomed-in view of the top 1,000 candidates detected by both metrics. For both metrics, we note that the observed agreement well exceeds random expectation.

To assess the comparative efficiency of each platform in detecting known splicing events, we compared the splicing events identified using our previously computed SI as well as a Student's t-test on the NI metrics. The analysis of SI values does not provide a statistical significance level, but simply ranks all exons by the magnitude of fold changes in the SI. The t-statistic takes into account the variance across replicates and ranks the candidate exons by the strength of statistical evidence. The choice of fold-change or statistical significance approaches to select top candidates in microarray studies has been heavily debated within the analytical community. Hence, we present the results of both methods. For a given number of top splicing candidates detected by each metric from each platform, we determine how many correspond to annotated Alt Events. It should be noted that: 1) the UCSC database may not be complete; 2) some events overlap each other with respect to its genomic locus; and 3) not all events in the database are expected to be variable across our two tissue types analyzed here. However, the over-representation (over random expectation) of events common to the UCSC database and the microarray results is a useful measure of cross-validation and correctness of the results. For both computed metrics, the Gene Array and Exon Array performed comparably (Figure 4). Within the top 1,000 candidate events, there is a three-fold enrichment, over random expectation, for events that are annotated within the Known Alt Events database. It should also be noted that this enrichment is consistently higher for SI analysis than for the t-test analysis, suggesting that candidates selected using the fold change method have a better true positives to false positives ratio, as compared to the statistical significance based selection.

**Figure 5:**
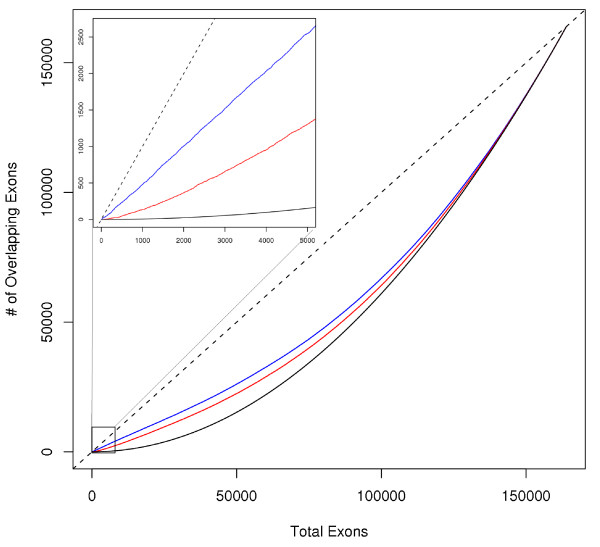
**Variability of detected splicing events across platforms**. The Gene Array and Exon Array show a modest amount of overlap of detected splicing events as measured using SI (blue) and t-test (red). However, both metrics perform visibly better than what is expected by random chance (black). As the total number of exons being considered increases, the overlap reaches saturation. The subplot provides a zoomed-in view of the top 5,000 candidates detected by both metrics. The dashed black line represents the x-y diagonal. Again, it should be noted that the lists produced using the fold-change method are more stable across platforms than the lists produced using the t-test.

The above analysis suggests that across the core probe sets common to both platforms, the Gene Array and the Exon Array have similar power and false positive rates in detecting alternative splicing. To determine whether the two platforms are in fact detecting the same events, we compared the inter-platform overlap. Briefly, we first considered all exons that are shared between the Gene Array and Exon Array (163,910 exons). Next, we independently ordered each list of exons (i.e. Gene Array and Exon Array exon lists) in absolute descending value for SI and decreasing p-value significance for t-test. Then, considering the top *n *candidates from each list, we counted the number of common exons found in both sample groups. This computation was repeated for increasing values of *n*. We found that overall, both metrics showed reasonable reproducibility among the top selection of exon candidates, not achieving perfect agreement but performing significantly above random expectation (Figure 5). Our results suggest that while both platforms identify annotated splicing events, there is some variability in what each platform preferentially detects.

## Discussion

The widespread occurrence of alternative splicing and tissue-specific transcript isoforms adds another layer of complexity to our understanding of human variation. Recent publications have estimated that as many as 95% of human multi-exon genes are influenced by alternative splicing [26,27] - a significant increase from past estimates of 74% [28]. In addition to custom designed isoform-sensitive microarrays [29], commercial technologies such as the Affymetrix GeneChip Human Exon 1.0 Array have made research in these areas more accessible. The most recent addition to the Affymetrix product line, the GeneChip Human Gene 1.0 Array, is a whole-transcript microarray designed to target the entire length of each gene with the purpose of optimizing gene expression profiling. However, the probe placement strategy of the Gene Array may also make it suitable for detection of alternative splicing. The goal of our study was to compare the performance of the Gene Array with the Exon Array and subsequently determine whether the Gene Array was capable of detecting differentially expressed isoforms. We mention two benefits that such knowledge would provide researchers. Firstly, the ability of older 3' targeting gene expression to detect alternative splicing and isoform variants was limited mainly due to probe placement. By contrast, the Gene Array interrogates the entire length of the gene and has been shown to be an excellent platform for measuring gene expression [12]. The whole-transcript design yields the potential for examining expression of individual exons and consequently, as already demonstrated in the well-studied Exon Array, the profiling of isoform variation. Second, while the Gene Array is more economical than the Exon Array, the study of alternative splicing can become more accessible by providing researchers an additional application to go along with gene expression studies.

We modelled our analysis approach after our previously successful comparative analysis of the Exon Array with other 3' arrays outlined in Bemmo et al. [11]. Our study was made possible by having datasets of both platforms assayed on the commercially available MAQC RNA samples, consisting of a high quality biological sample set derived from the human brain and human universal reference. The MAQC is ideal and valuable for benchmarking purposes and for detection of transcript isoform variation, due to the high degree of alternative splicing that occurs in brain tissue.

We first compared the platforms at the gene expression level and concluded that the Gene Array results are highly concordant with the Exon Array results, reaffirming the utility of the Gene Array as a gene expression profiling tool. Noting that the majority of discordant genes between the two platforms were weakly expressed, we used a detection threshold cutoff to filter and correct for these genes. Interestingly, the correlation gradually decreased as the threshold increased past 30, our selected optimal cutoff. With our improved correlation results, we were encouraged to consider comparisons at the exon level.

Studying the Gene Array at the exon level posed a significant challenge as no known approach to summarize probe hybridization intensities to reflect exon expression has been developed for the Gene Array. To overcome this, we modified the Gene Array's Probe Group File such that the probe groupings correspond roughly to an exon rather than a gene. The modified Gene Array groups could then be subjected to the same PLIER summarization step as the Exon Array data. This resulted in full summarization of probe sets consisting of two or more probes, and simple normalization and background correction for single-probe groupings. This approach makes the Gene Array and Exon Array analyses directly comparable. We have made available this modified file for download in Additional File 5.

An interesting question that was raised in this study was whether the reduced probe coverage could sufficiently profile exon expression levels. We first observed that the majority of the exons were targeted by one or two probes on the Gene Array, and that of these, the calculated fold changes within our datasets were highly correlated with those on the Exon Array. A similar result was observed when the number of probes per probe set was reduced on the Exon Array and re-summarized. As the majority of the Gene Array probes were generally selected for consistency with the Exon Array as well as uniquely matching to the human genome, we would expect that the Gene Array to be optimized for effective probe hybridizations. This suggests that in general exon expression levels on the Gene Array may be sufficiently estimated with fewer than four probes.

To shed light on the potential application of the Gene Array to detect transcript isoform variation, we used two previously described genes, *ELAVL1 *and *MADD*, as examples for comparison. The Gene Array demonstrated high reproducibility in exon expression levels and detected the same splicing events as the Exon Array, as seen by their overall similar fold change pattern when visualized on a plot. Interestingly, in the case of *ELAVL1*, the gene level fold changes were not in agreement. Here, the long isoform was targeted by twice as many probe sets than the short isoform. Since half of these interrogated the extended 3' region of the isoform, the overall gene level expression summarization as calculated by PLIER is heavily influenced by the individual expression measurements of its probes. In this context, gene level fold changes are not meaningful in describing isoform events. Despite these differences, visualization of the exon level probe sets provided supporting evidence that *ELAVL1 *contains differentially expressed isoforms. This reiterates the importance of using careful visualization to examine exon level expression for isoform variation, as previously noted by Bemmo et al. [11]. Such findings can potentially be of significant biological value that may warrant further research.

To better understand the splicing detection differences, we used splicing index analysis to compare the performance of the two platforms. Variations or inaccuracies in expression estimates can have a large impact on the SI, making direct comparisons of such values to determine inter-platform reproducibility a challenge. However, from our analysis we conclude that the isoform-level results, as determined using SI analysis, have a good degree of correspondence between platforms. This further suggests that on a whole-genome scale, the Gene Array may be a valuable tool for profiling alternative splicing.

We note two limitations of the Gene Array. Firstly, with the exception of the analysis on detecting known splicing events illustrated in Table 1, we considered only the probes that were matched with the Exon Array. This ensures that we are considering the same Exon Array probe set genomic boundaries. It should be noted that the probes unique to the Gene Array that were omitted may provide informative expression data. However, their inclusion would require determining whether they can be accurately mapped within an exon region defined by an external annotation source, which we did not do for the sake of this comparative analysis. In addition, in order to maintain high confidence results, speculative Exon Array probe sets not from the core design were excluded in this part of our analysis. Secondly, the Gene Array is less comprehensive than the Exon Array as it mainly targets only well-annotated genes. As a result, the Gene Array may be limited in its potential to identify novel isoforms in genes that have not been well-studied and/or annotated by database curators. As expected, from comparing the probe locations of the two platforms with the Known Alt Events track on the UCSC Genome Browser, the Exon Array targets a higher proportion of annotated events. Notably, by further comparing the results of the MAQC data analysis and the Alt Events database, both platforms demonstrate the ability to effectively detect these known splicing events, with comparable false positive rates.

Our approach for exon level summarization on the Gene Array relies on utilizing existing Affymetrix software (i.e. PowerTools) and analysis pipelines. As it simply involves the replacement of a standard annotation file, it is relatively easy for users to make their own modifications. Users are also free to supply their own parameter settings for the summarization step to suit their analytical needs.

In addition to microarray studies, an emerging technology in genomics is next-generation sequencing. In particular, high-throughput RNA sequencing (RNA-Seq) enables the profiling of the entire transcriptome and produces both sequence and gene expression information. RNA-Seq holds a number of advantages to microarray solutions including more precise expression measurements with fewer biases and the ability to discover novel transcripts and isoforms [30,31]. While the cost of this technology is rapidly falling, currently they are still considerably more expensive than microarrays. We expect to see microarrays to continue to be in use and look forward to RNA-Seq adding greater power to transcriptome analysis.

We also note that while this manuscript was under review, another method for differential splicing detection using the Gene Array was published [32]. This provides further support for Gene Arrays to be potentially used as a cost-effective platform for alternative splicing discovery.

## Conclusion

To summarize, technological advancements in whole-transcript platforms have become new standards for microarray expression analysis studies. Using a modified probe set annotation library file, exon expression summarization is made possible on the Gene Array and demonstrates high concordance with the Exon Array as well as the potential to detect tissue-specific transcript isoforms. While not as comprehensive as the Exon Array, the Gene Array overlaps a good proportion of known splicing events (46% vs. 75%) which it may have the potential to detect, particularly cassette exons. We would also like to point out that the Gene Array (as analyzed here) uses a considerably simpler processing method than the Exon Array (100 ng of total RNA versus 1 μg, and no ribosomal RNA reduction step), and is considerably less expensive. We hope our findings will be of value for researchers using the Gene Array to profile gene expression and will be persuaded to investigate potential splicing and isoforms differences within their datasets, at no extra cost.

## Methods

### Data Acquisition

The MAQC raw data (.CEL files) for the Gene Array experiments and Exon Array experiments was downloaded from the NCBI Gene Expression Omnibus (GEO - http://www.ncbi.nlm.nih.gov/geo/) repository under the GEO records GSE9819 and GSE13072, respectively. Annotation and library files for both platforms were based on the March 2006 human genome assembly (UCSC hg18, NCBI Build 36) and downloaded from the Affymetrix website (http://www.affymetrix.com).

### Gene array and exon array hybridization

MAQC Universal Human Reference RNA (Stratagene) and Human Brain Reference RNA (Ambion) were used for both Affymetrix GeneChip Human Exon 1.0 ST and GeneChip Human Gene 1.0 ST arrays. For complete details, please refer to Bemmo et al. [11] and Pradervand et al. [12] for Exon Array and Gene Array MAQC studies, respectively. While both studies used similar protocols, we note that the ribosomal RNA reduction step was not performed and 100 ng instead of 1 μg was used for the Gene Array hybridization by Pradervand et al. [12]. While the complete Exon Array dataset contained experiments performed at two different sites, only those conducted at the McGill University and Genome Quebec Innovation Centre were considered for this analysis. To maintain consistency, four out of five Exon Array technical replicates per tissue group were arbitrarily selected to match the same number of replicates available in the Gene Array data.

### Data pre-processing and analysis

Processing of the data follows a similar procedure as described in Bemmo et al. [11]. The Affymetrix PowerTools software package was used to apply quantile normalization on the probe hybridization intensities and a probe logarithmic intensity error (PLIER) method [13] to summarize exon level and gene level expression intensities. The summarized values from all four replicates of each tissue group and each platform were combined by calculating the geometric mean. Pairwise correlation of the fold changes between the two platforms were computed using Pearson's correlation coefficient. For analysis on the Exon Array, we only used core probe sets that are supported by the most reliable and accurate annotation.

To perform exon level analysis on the Gene Array, we computationally modified the Probe Group File (PGF), a library file utilized by Affymetrix software, such that probes were grouped according to their corresponding exons rather than their gene. To ensure that the exon boundaries in these new groupings are consistent with the Exon Array probe set boundaries, we limited our analysis to a subset of common probes between both platforms as determined by concordant genomic chromosomal coordinates. This resulted in 409,775 (53.6% of 764,885 total Gene Array probes) common core probes that would be grouped based on Exon Array probe set definitions. For the sake of comparison, we gave these new exon groupings the same Exon Array probe set identifiers.

### Background filtering and correction of low expression signals

To filter out low expression signals which may represent noise or poorly hybridized probes, we examined whether the mean hybridization intensities of the brain and reference RNA samples across both platforms were below a threshold cutoff *c *(for a total of 4 mean intensities). If all four intensities were below *c*, then the gene would be discarded from the analysis. On the other hand, if at least one of the samples across both platforms were above *c*, then the gene would be retained but converted such that all below-*c *intensities are corrected to *c*. We performed an optimization of this filtering approach for a range of values for *c *and determined its effect on the correlation, number of discarded genes, and number of outliers. This optimization of threshold values yielded a desirable cutoff of 30 that maximized inter-platform correlation.

### Splicing index analysis

The splicing index (SI) provides a metric for comparing exon inclusion levels between two samples while taking into account for differential expression at the gene level. First, the normalized exon intensity (NI), which represents the ratio of the exon probe set intensity to the gene level intensity, is determined. The SI is then calculated by taking the log_2 _ratio of the NI from the two samples.

For splicing index analysis, we altered our filtering strategy in order to retain only genes that were expressed above background in both tissues. This more stringent filtering step is warranted since, by definition, only genes that are expressed in both samples can be differentially spliced across the samples. As in the exon level analysis, we then discarded all exons that were not expressed above background in at least one of the samples.

### Targeting known alternative splicing events

The Known Alt Events annotation track was downloaded from the UCSC Genome Browser database (human hg18; http://genome.ucsc.edu), which contained a total of 74,059 events. The overlap of each Alt Event with Gene Array probes and Exon Array probe sets was computed based on genomic coordinates. For the purpose of this analysis, all probe sets from both platforms were considered regardless of their evidence level (core, extended, or full). Coordinates that overlapped by at least one base pair were considered an overlap. We used this liberal definition in order to capture all possible overlaps. Probes that may share even the slightest overlap may be expected to show a detectable change in expression.

### Detection of common splicing events between platforms

We aimed to determine whether the two platforms tended to detect the same alternative splicing events, or whether perhaps they preferentially exhibited optimal sensitivity to detect different candidate events. We produced lists of top *n *candidate alternative splicing events for each platform and determined the overlap between the two lists. To evaluate whether or not the observed inter-platform reproducibility was due to chance, we calculated the random expectation of observing an overlap by applying the equation:

where *m *is the total number of overlapping exons in the entire dataset, and *f*_*GA*_*(n) *and *f*_*EA*_*(n) *are the frequencies of randomly selecting *n *candidate exons from the Gene Array and Exon Array, respectively. In this case, since we limited our analysis to only exons that are common in both platforms, *m *is 163,910 and *f*_*GA*_*(n) *= *f*_*EA*_*(n) *= *n*/163,910, which simplifies the equation to *E*_*overlap *_= *n*^2^/163,910.

## Authors' contributions

KCH performed the computational analysis, prepared the figures and wrote the manuscript. JCH performed parts of the alternative splicing analysis. JM conceived the study and provided feedback throughout the project. All authors have read and approved the final manuscript.

## Supplementary Material

Additional file 1 **Optimization results for background correction analysis**. We carried out an optimization analysis using a range of expression cutoff values to determine the optimal correlation between log_2_-transformed fold changes detected by the Gene Array and Exon Array. This computation was performed at the gene level and exon level for an arbitrary range of cutoff values between 0 and 60. The results are plotted for both the (Figure S1A) gene level and (Figure S1B) exon level. The correlation is indicated by the red line while the number of total genes retained is indicated by the blue vertical bar plots. Click here for file

Additional file 2 **Comparison of exon level fold changes according to the number of probes per exon probe set on the Gene Array**. After summarizing the Gene Array exon expression levels, the 205,465 exons were grouped according to the number of probes targeting each exon probe set. The fold changes in each subset were then compared to its counterpart on the Exon Array. Click here for file

Additional file 3 **Effect of reducing the number of probes per probe set on the Exon Array**. Probes were randomly removed from each probe set on the Exon Array. The exon expression values were re-summarized using PLIER and the recalculated fold changes were compared with the full Gene Array results. This process was repeated a second time to yield another set of results. The analysis was subjected to the same expression filtering criteria, resulting in a varying number of probe sets in each case. Click here for file

Additional file 4 **Intra-platform comparison of SI values**. Correlation plots comparing SI values at the intra-platform level, one for (Figure S4A) Gene Array and one for (Figure S4B) Exon Array. Two sets of replicates per tissue group (i.e. two for brain and two for reference) were randomly selected and compared to the remaining two sets replicates. The analysis was subjected to the same filtering criteria as in Figure 3. Click here for file

Additional file 5 **Gene Array Probe Group File modified for exon level expression analysis**. We provide the modified PGF file which consists of only Gene Array probes from the Exon Array subset, representing 205,465 probe sets. The new exon groupings use the same Exon Array probe set identifiers. Furthermore, the mappings have been done only for the core Exon Array probe sets. Click here for file
