# Supplementary figures and images for: Comparison of Affymetrix Gene Array with the Exon Array shows potential application for detection of transcript isoform variation
Source: BMC Genomics. 2009 Nov 12;10:519. doi: 10.1186/1471-2164-10-519 (PMC2780461; doi:10.1186/1471-2164-10-519)

**Figure S1A – Gene-level**

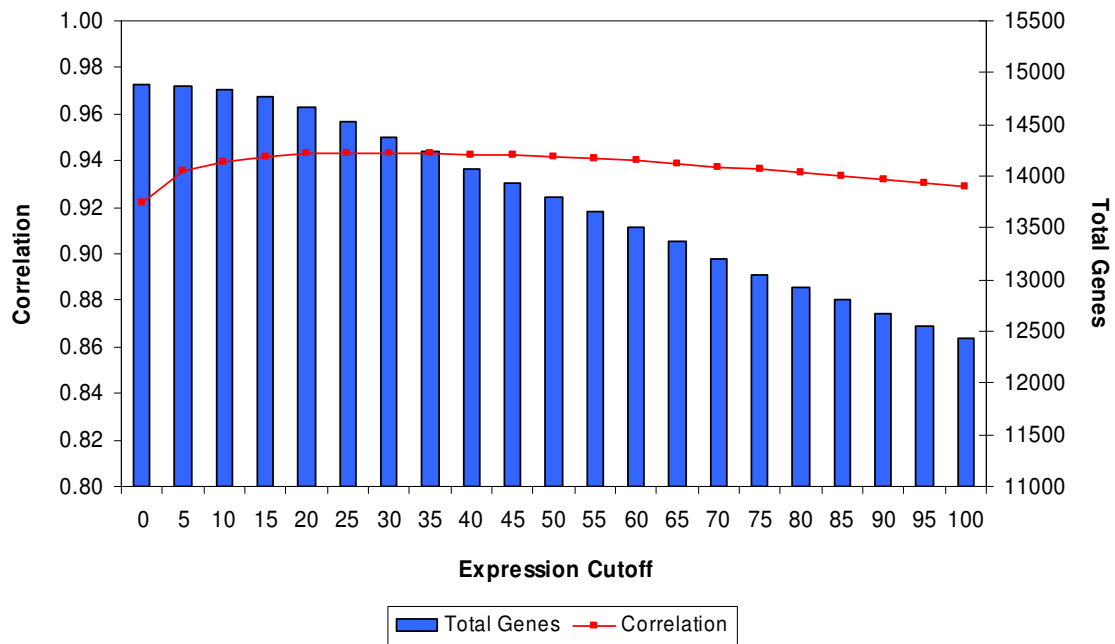

**Figure S1B – Exon-level**

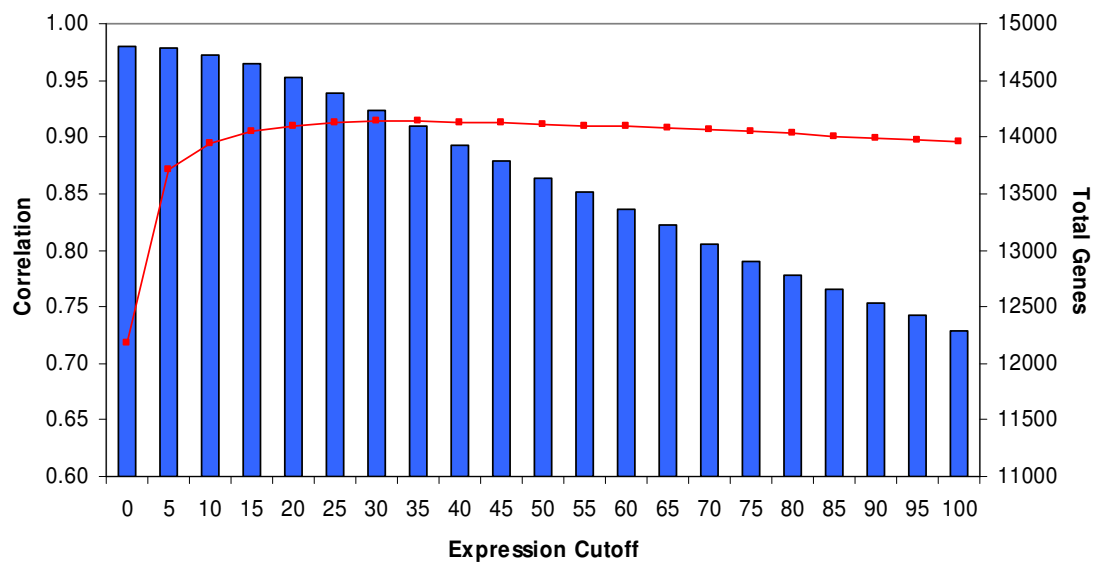

Supplement: Additional file 1 — Optimization results for background correction analysis. We carried out an optimization analysis using a range of expression cutoff values to determine the optimal correlation between log2-transformed fold changes detected by the Gene Array and Exon Array. This computation was performed at the gene level and exon level for an arbitrary range of cutoff values between 0 and 60. The results are plotted for both the (Figure S1A) gene level and (Figure S1B) exon level. The correlation is indicated by the red line while the number of total genes retained is indicated by the blue vertical bar plots. [file 1471-2164-10-519-S1.pdf]

**Figure S4A – Gene Array vs. Gene Array**

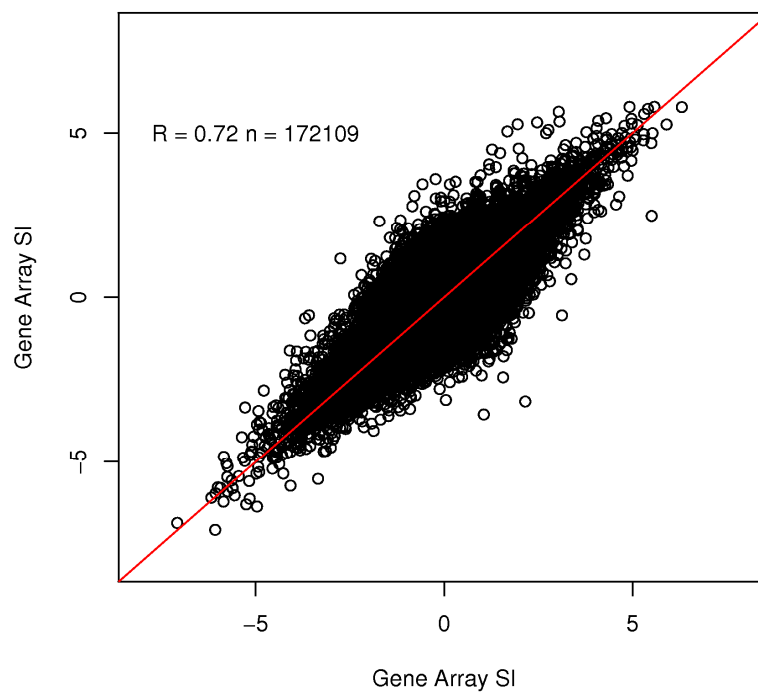

**Figure S4B – Exon Array vs. Exon Array**

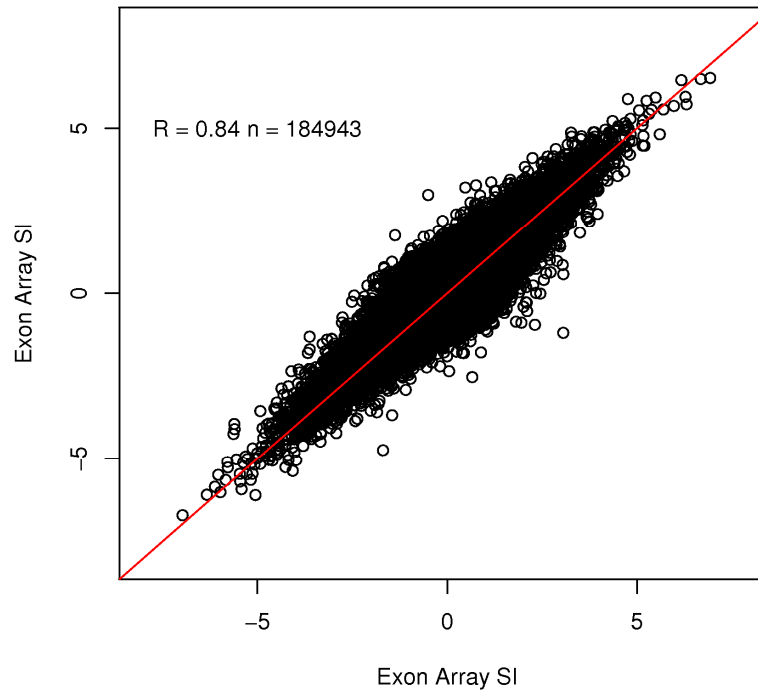

Supplement: Additional file 4 — Intra-platform comparison of SI values. Correlation plots comparing SI values at the intra-platform level, one for (Figure S4A) Gene Array and one for (Figure S4B) Exon Array. Two sets of replicates per tissue group (i.e. two for brain and two for reference) were randomly selected and compared to the remaining two sets replicates. The analysis was subjected to the same filtering criteria as in Figure 3. [file 1471-2164-10-519-S4.pdf]
